# Caffeic acid phenethyl ester promotes palatal wound healing and enhances wound-associated macrophage CD68 expression

**DOI:** 10.1016/j.jtumed.2025.07.010

**Published:** 2025-08-08

**Authors:** Arin O. Suningdyastiningrum, Islamy R. Hutami, Yuli Berliani, Yayun S. Rochmah

**Affiliations:** a Master Program of Dental Sciences (Oral Biology), Faculty of Dentistry, Universitas Islam Sultan Agung, Indonesia; b Department of Orthodontics, Faculty of Dentistry, Universitas Islam Sultan Agung, Indonesia; c Department of Oral Surgery, Faculty of Dentistry, Universitas Islam Sultan Agung, Indonesia

**Keywords:** إسترفينيثيلحمضالكافيين؛, الحنك؛, الخلاياالبلعمية؛, الكلماتالمفتاحية, تيجياف-بيتا١, سيدي-٦۸؛, Caffeic acid phenethyl ester, CD68, Macrophages, Palatal, TGF-β1

## Abstract

**Objectives:**

Palatal wounds may result in open lesions, which can lead to extended inflammation, discomfort, wound contraction, scar tissue development, interference with phonation or mastication, and disruption of maxillofacial growth. Flavonoids are known to possess the ability to diminish macrophage pro-inflammatory activity and to facilitate macrophage-mediated resolution of inflammation, thereby accelerating the healing process. Caffeic acid phenethyl ester (CAPE), the principal bioactive constituent of propolis, belongs to this family, and is known to diminish inflammatory cell count, accelerate wound contraction, and promote re-epithelialisation by decreasing lipid peroxidation and the production of reactive oxygen species. This study assesses the impact of CAPE on macrophage expression markers during palatal wound healing.

**Methods:**

A total of 45 male BALB/c mice, aged 3 months, were allocated into control (saline), CAPE-treatment (CAPE-T), and CAPE-pretreatment (CAPE-PT) groups, and underwent palatal tissue excision at the mid-hard palate. CAPE was administered intraperitoneally at a dosage of 4 mg/kg of body weight. Clinical observation of palatal wound closure and liver were conducted by hematoxylin-eosin (HE) analysis. Immunohistochemical (IHC) examination of CD68 and TGF-β1 was conducted to assess the inflammatory markers during palatal wound closure.

**Results:**

On days 3 and 5, the CAPE-T and CAPE-PT groups showed faster wound healing than the control. CAPE also caused a significant weight gain in mice. IHC analysis revealed more CD68-positive cells and higher TGF-β1 levels, along with lower TNF-α and iNOS expression on day 5.

**Conclusion:**

CAPE positively influences palatal wound healing by accelerating wound closure and modulating CD68, TGF-β1, TNF-α, and iNOS expression.

## الملخص

**أهداف البحث:**

قد تسبب جروح الحنك آفات مفتوحة، مما قد يؤدي إلى التهاب ممتد، وانزعاج، وتقلص الجرح، ونمو نسيج ندبي، وتداخل في النطق أو المضغ، واضطراب في نمو الوجه والفكين. من المعروف أن الفلافونويدات تمتلك القدرة على تقليل نشاط الخلايا البلعمية المحفز للالتهابات، وتسهيل شفاء الالتهاب بوساطة الخلايا البلعمية، مما يسرع عملية الشفاء. ينتمي إستر فينيثيل حمض الكافيين، المكون الحيوي الرئيسي في العكبر، إلى هذه الفصيلة، ومن المعروف أنه يقلل من عدد الخلايا الالتهابية، ويسرع تقلص الجرح، ويعزز إعادة التئام الظهارة عن طريق تقليل بيروكسيد الدهون وإنتاج أنواع الأكسجين التفاعلية. تقيم هذه الدراسة تأثير إستر فينيثيل حمض الكافيين على علامات تعبير الخلايا البلعمية أثناء التئام جروح الحنك.

**طرق البحث:**

تم توزيع ٤٥ فأرا ذكرا من نوع "سي/بالب" بعمر ٣ أشهر، على مجموعتين: مجموعة التحكم (المحلول الملحي)، ومجموعتي المعالجة بـ إستر فينيثيل حمض الكافيين، ومجموعة المعالجة المسبقة بـ إستر فينيثيل حمض الكافيين، وخضعوا لاستئصال أنسجة الحنك من منتصف الحنك الصلب. أعطي إستر فينيثيل حمض الكافيين عن طريق الحقن البريتوني بجرعة ٤ ملغم/كغم من وزن الجسم. أجريت المراقبة السريرية لإغلاق جرح الحنك والكبد من خلال تحليل الهيماتوكسيلين-إيوزين. أجري فحص مناعي كيميائي لـ "سي دي-٦۸" و "تي جي اف-بيتا١" لتقييم العلامات الالتهابية أثناء إغلاق جرح الحنك.

**النتائج:**

في اليومين الثالث والخامس، أظهرت مجموعتا المعالجة بـ إستر فينيثيل حمض الكافيين ومجموعة المعالجة المسبقة بـ إستر فينيثيل حمض الكافيين سرعة في التئام الجروح مقارنة بالمجموعة الضابطة. ومع ذلك، أدى إعطاء إستر فينيثيل حمض الكافيين أيضا إلى زيادة ملحوظة في وزن الفئران. كشف تحليل الفحص المناعي الكيميائي عن زيادة ملحوظة في الخلايا الإيجابية لـ "سي دي-٦۸" في كل من مجموعتي المعالجة بـ إستر فينيثيل حمض الكافيين ومجموعة المعالجة المسبقة بـ إستر فينيثيل حمض الكافيين مقارنة بالمجموعة الضابطة، إلى جانب ارتفاع مستويات "تي جي اف-بيتا١" وانخفاض في التعبير عن "تي ان اف-ألفا" و "آي ان او اس" في اليوم الخامس بعد الجرح.

**الاستنتاجات:**

يؤثر إستر فينيثيل حمض الكافيين إيجابيا على التئام الجروح الحنكية من خلال تسريع التئام الجروح وتعديل التعبير عن "سي دي-٦۸" و "تي جي اف-بيتا١" و "تي ان اف-ألفا" و "آي ان او اس".

## Introduction

Palatal wounds, whether resulting from trauma or surgical procedures, often exhibit delayed healing due to the oral cavity's constant exposure to microbial flora and mechanical stress. Such wounds can lead to prolonged inflammation, pain, dietary modifications, and functional impairments, including difficulties in mastication and phonation. Moreover, disruption of the epithelial barrier increases the risk of infection, bone exposure, and chronic, non-healing wounds.^1^, ^2^, ^3^, ^4^

Wound healing is a complex process comprising four overlapping phases: haemostasis, inflammation, proliferation, and maturation. Macrophages play a pivotal role in this process, initially activating a pro-inflammatory (M1) phenotype that facilitates wound debridement through the production of nitric oxide (NO), reactive oxygen species (ROS), and inflammatory cytokines, such as interleukin (IL)-1, IL-6, and tumour necrosis factor-alpha (TNF-α). As healing progresses, monocyte-derived macrophages secrete transforming growth factor-beta (TGF-β1), inducing a phenotypic switch to the anti-inflammatory (M2) macrophage subtype, which promotes fibroblast proliferation, keratinocyte migration, and tissue remodelling.^5^, ^6^, ^7^, ^8^, ^9^, ^10^, ^11^, ^12^

Caffeic acid phenethyl ester (CAPE) as bioactive flavonoid derived from propolis, possesses anti-inflammatory and antioxidant effects that aid wound healing. CAPE modulates macrophage activity by attenuating pro-inflammatory responses and promoting M2 polarisation, thereby accelerating tissue regeneration.^13^, ^14^, ^15^, ^16^, ^17^, ^18^ Additionally, CAPE has been shown to reduce inflammatory cell infiltration, enhance wound contraction, and facilitate tissue formation.^16^^,^^19^, ^20^, ^21^ Several studies have explored the potential of CAPE in palatal wound healing, demonstrating its positive effects on re-epithelialisation and tissue repair.^22^^,^^23^ Moreover, natural compounds such as CAPE often exhibit fewer adverse effects compared to synthetic pharmaceuticals, making them promising candidates for therapeutic applications.^24^

While research has investigated CAPE's role in palatal wound healing, the underlying mechanisms—particularly its influence on macrophage activity—remain inadequately characterised. This study aims to further elucidate CAPE's effects on palatal wound healing in a murine model, with a specific focus on its regulation of macrophage markers such as CD68, TGF-β1, TNF-α, and iNOS. By advancing our understanding of CAPE's immunomodulatory properties, this research seeks to contribute to the development of novel therapeutic strategies for optimising oral wound healing.

## Materials and methods

### Animal models

This study was approved by the Health Research Ethics Commission Faculty of Dentistry, Universitas Islam Sultan Agung, Indonesia (protocol No. 573/B.1-KEPK/SA-FKG/VI/2024). Twelve-week-old male BALB/c mice (total: 45) weighing an average of 30 g were used. The mice were kept in standard cages lined with wood husk. The environment was maintained at 24 °C, with a 12-h light/dark cycle, and the mice had free access to drinking water and a food pellets.^25^ The body weights of the mice were measured every 24 h. Each cage was given 20 g and the mice have free access of food.^26^

**Figure 1:**
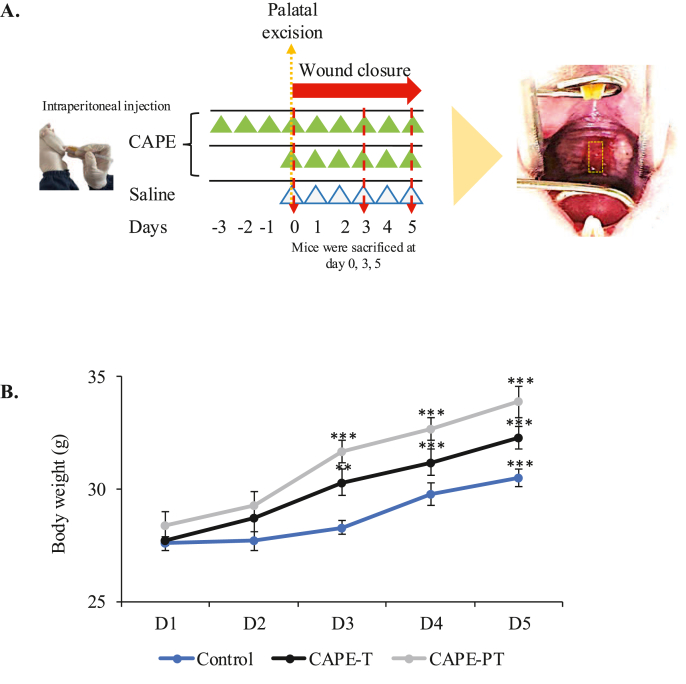
**Effect of CAPE on Metabolism**. (A) BALB/c mice were randomly assigned to a control group, a pre-treatment group (CAPE-PT), or a treatment group (CAPE-T). The control group received intraperitoneal injections of normal saline on the indicated days (white triangle). The pre-treatment group received intraperitoneal injections of CAPE-PT starting three days before the wound was created and continuing until the fifth day post-wound (green triangles, upper row). The CAPE-T group received intraperitoneal injections of CAPE beginning on the day the wound was created and continuing until the fifth day post-wound (green triangle, lower row). On day zero a full-thickness excisional wound was created in the hard palate. Palatal wound healing was evaluated on days 0, 3, and 5 by dissecting euthanised mice after a full-thickness excisional wound (approximately 2.0 mm in width) was created in the hard palate. (B) Body weight was measured from day 1 to day 5 following palatal wound creation. Data are presented as mean ± SD. Data are presented as mean ± SD. ∗∗∗p < 0.001, ∗∗p < 0.01.

All attempts, including anesthesia, were taken to minimize the experiment from pain. Mice were euthanised by cervical dislocation following chloroform exposure zero, 3, or 5 days after wound creation.^25^^,^^27^ Ready-made CAPE (Sigma–Aldrich, St Louis, MO, USA) was dissolved and diluted in saline. The dose of CAPE used was 4 mg/kg,^28^^,^^29^ administered intraperitoneally (i.p.) every 24 h according to a schedule depending on their control group (Figure 1A). Systemic administration via i.p. injection has been widely employed in preclinical studies to evaluate broader pharmacological effects on wound healing, including in the oral cavity. Previous studies have successfully utilized i.p. administration to assess CAPE's role in palatal wound healing.^22^^,^^30^ Systemic delivery enables the evaluation of key mechanisms such as inflammatory modulation, antioxidant activity, and cytokine regulation, all of which contribute to wound repair.^19^^,^^29^

### Preparation of wound

To create the wound, mice were anesthetised with a cocktail of 90–150 mg/kg ketamine hydrochloride and 7.5–16 mg/kg xylazine hydrochloride via intramuscular injection.^25^ Once anesthetised, a full-thickness wound excision was performed on the hard palate using a modified surgical blade number 11, creating a wound 2.0 × 3.0 mm^2^ in size.^27^^,^^31^^,^^32^To minimise variability, all wounds were created by the same operators (AOS, YB) under magnification. However, slight variations in initial wound size may still be present, which is acknowledged as a study limitation.

Mice were randomly assigned into three groups: control, CAPE treatment (CAPE-T) and CAPE pretreatment (CAPE-PT), with 15 animals per group. This is a blind study: in all procedures described below, examiners were unaware of the origin of the sample.

Palatal wound tissue samples were clinically and histologically examined. The detailed experimental timeline is depicted in Figure 1A. High-resolution photographs of the palatal wounds and liver were captured immediately after euthanasia on days 0, 3, and 5 using a digital microscope equipped with a macro lens (Digital Microscope 1600x Zoom Magnifier, MTA-169929798). The wound area measurements were performed by a single masked and calibrated examiner using ImageJ software (ImageJ 1.4v for Mac OS, National Institutes of Health, Bethesda, MD).^25^^,^^33^^,^^34^

### Hematoxylin and eosin (HE) histological analysis

The histological analysis of wound distance is based on digital photos. To prepare for histological examination, palatal tissues were fixed in 4 % paraformaldehyde (PFA) for 48 h and decalcified in 14 % EDTA/PBS for 20 days. The tissue section was then trimmed and embedded in paraffin wax after dehydration. Serial transverse sections were cut in 5 μm thickness, then dewaxed and rehydrated conventionally. These sections were stained with HE to analyze wound closure, especially the epithelium-edges distance (EED). The images were captured using a microscope (Olympus, CX21, Japan) and image-capture software. The wound's biggest central diameter was used to measure EEDs using imageJ.^25^^,^^27^^,^^35^

The percentage of wound closure for each animal at each time point were measured using the formula: % Wound Cloure: [1 – (wound area)/(original area)] x 100 %^36^**.** In this study, all wound area measurements were performed by a single calibrated examiner who was blinded to the sample groups. To ensure consistency, the examiner re-evaluated 20 % of the wound images at two different time points, at least one week apart. The measurements showed minimal variation, indicating a high level of consistency.

For the mice euthanised on day 0 or day 5, the livers were also collected for histological analysis. The liver tissues were fixed in 4 % PFA over 48 h, dehydrated by graded ethanol, then embedded in paraffin. Sections with a thickness of 5 μm were prepared and stained with HE.^34^ The images were captured using a microscope and imageJ capture software.

### Immunohistochemistry (IHC) assay

IHC kits (Specific HRP/DAB Detection IHC Kit, UK, ab64264) were used with antimouse monoclonal CD68 (Santa Cruz Biotechnology, sc-20060, TX, USA), antirabbit polyclonal TGF-β1 (Elabscience Biotechnology, E-AB-70076, Hubei, China), antimouse monoclonal TNF-α (Santa Cruz Biotechnology, sc-52746, TX, USA), and antimouse monoclonal iNOS (ThermoFisher Scientific, MA5-17139, MA, USA) as primary antibodies. After 15 min in a slide heater, tissue slices were deparaffinized three times in 5 min with fresh xylene and dehydrated with absolute alcohol. The peroxide block was administered at room temperature for 15 min to inhibit endogenous peroxidases. After removing the block with distilled water, the sample was washed with citrate buffer (pH 6.0) for 10 min.^34^^,^^35^

After removing the excess solution, the sections were incubated with the primary antibody, the sections were then incubated with the anti-mouse or anti-rabbit secondary antibody (ELK Biotechnology) for 1 h at room temperature, followed by two consecutive, 5-min buffer washes. Before applying to sections, one drop of chromogen diaminobenzidine was combined with 1 ml of buffer in a mixing vial; this was then added over the sections. After 5 min, the sections were washed first with the buffer, then by water, and then counterstained with Harris hematoxylin. Finally, the section was air dried, cleared, and mounted with xylene.^35^^,^^37^ The images were captured using a microscope. The cells with positive CD68, TGF-β1, TNF-α, and iNOS expression were then counted. A blinded professional pathologist evaluated the cells without knowledge of the experimental conditions. To ensure accuracy and reliability in the histological assessment, cell counts were performed three times per sample across five fields of view, and the average value was calculated.

### Statistical analysis

Statistical analyses were conducted using SPSS (IBM SPSS Statistics 26, Stanford University). Prior to hypothesis testing, a power analysis was performed to determine the appropriate sample size for detecting significant differences among groups, ensuring the ethical use of animals in accordance with the replacement, reduction, and refinement (3Rs) principle.^38^^,^^39^ The normality of data distribution was assessed using the Shapiro–Wilk test. For normally distributed data, one-way analysis of variance (ANOVA) followed by Tukey's honest significant differences (HSD) post hoc test was applied to assess intergroup differences. A p-value of less than 0.05 was considered statistically significant.

## Results

### Effects of CAPE on body metabolism

During the experimental period, the animals showed good post-wound recovery, with no signs of pain or distress. Mice in the CAPE treatment group exhibited a significant increase in body weight compared to the control group (Figure 1B). Clinical observation and hematoxylin and eosin (H&E) staining of the liver revealed no noticeable differences in appearance between the groups (Supplementary 1A). Measurements of liver size trended slightly upward with CAPE exposure but showed no significant differences across groups (Supplementary 1B). Additionally, macrophage positive cell counting in the liver did not reveal macrophage presence in any group (Supplementary 1C). These findings suggest that CAPE administration via intraperitoneal injection may influences metabolic processes, leading to increased body weight and liver size without affecting macrophage infiltration in the liver.

### Effects of CAPE on palatal wound healing

**Figure 2:**
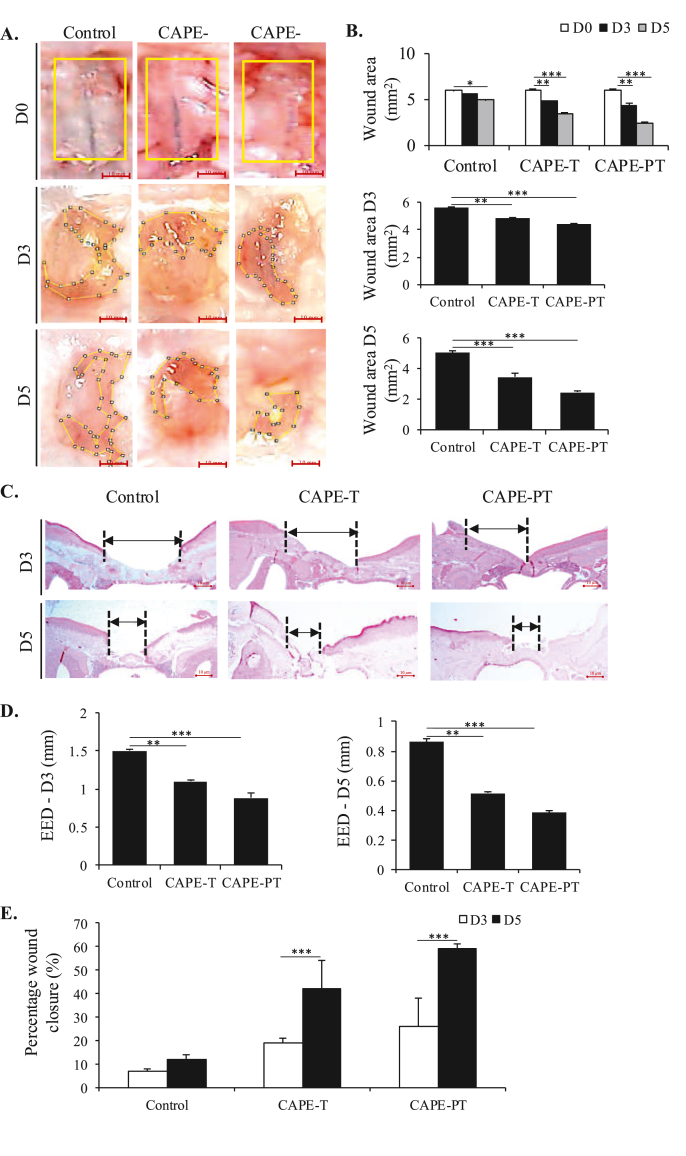
**Effect of CAPE on Palatal Wound Healing.** (A) Images of the palatal wound area obtained on days 0, 3, and 5. Representative images from five mice per group are shown (scale bar: 10 mm), with the wound perimeter outlined in yellow. (B) Measurement of wound areas on days 0, 3, and 5 after palatal wound creation. Data are presented as mean ± SD with five mice per group. (C) HE staining was performed to analyze the EED on days 3 and 5 (scale bar: 100 μm). Representative images are shown. (D) EED measurements of palatal tissue on days 3 and 5. (E) Measurement of palatal wound closure percentage on days 3 and 5. Data are presented as mean ± SD. ∗∗∗p < 0.001, ∗∗p < 0.01, ∗p < 0.05.

Clinical observations of palatal wound healing were conducted, with wound area measurements. On days 3 and 5, the control group showed statistically significant differences in wound area compared to the treatment and pre-treatment groups (Figure 2A, B). Although the differences between the treatment and pre-treatment groups were not significant on either day, the latter consistently had the smallest wound areas, suggesting a potential advantage pre-treatment.

Histomorphometric analysis of EED from HE staining showed a similar pattern to wound area when comparing the control, treatment, and pre-treatment groups. EED measurement of wound closure in the control group were significantly different from both the treatment and pre-treatment groups, on the 3rd and 5th days (Figure 2C, D). While there were no significant differences between the treatment and pre-treatment groups, the pre-treatment group had shorter EED values, indicating more efficient wound closure at all time points. The percentage of wound closure analysis showed that the CAPE-T and -PT groups showed significantly greater percentage of wound closure compared to the control group on the 3rd and 5th days (Figure 2E). Overall, the analysis of wound area closure and EED suggests that systemic administration of CAPE can enhance palatal wound healing, with pre-treatment showing the most favourable outcomes in wound area reduction and faster epithelialisation.

### Effects of CAPE on CD68, TGF-β1, TNF-α, and iNOS expression

**Figure 3:**
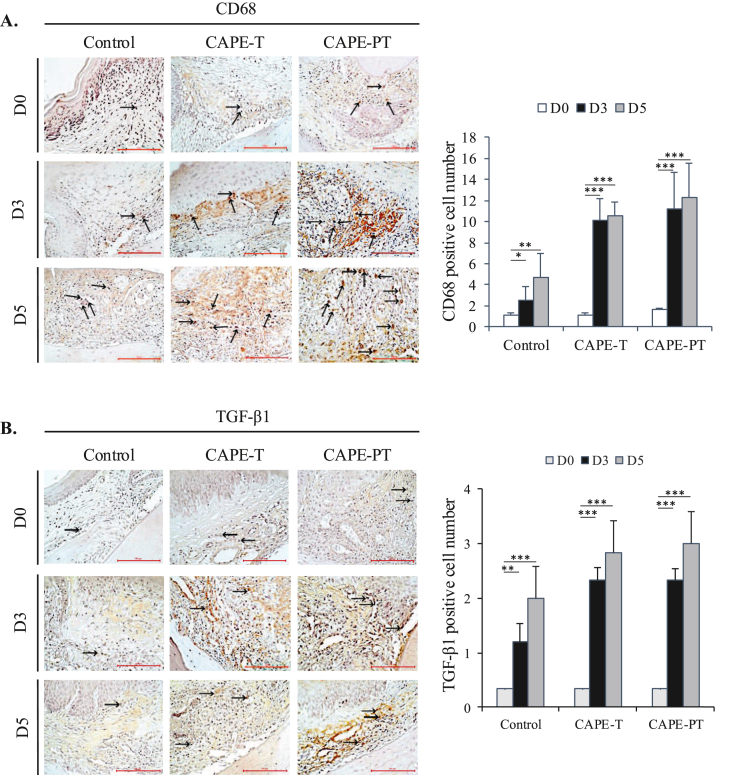
**Expression of IHC Biomarkers CD68 and TGF-β1 in Palatal Wound Healing.** (A) Representative IHC images and counting of CD68-positive cells in the perimeter of the wound site. (B) Representative IHC images and quantification of TGF-β1-positive cells at the wound site. Data are presented as mean ± SD. ∗∗∗p < 0.001, ∗∗p < 0.01, ∗p < 0.05.

In Figure 3A we seen an example section from each category (group, day) and the mean number of CD68-positive cells detected in the category. The control group exhibited the lowest number of CD68-positive cells at all time points. In addition, the difference between the control group and the two CAPE groups was significant at all time points. The CAPE-PT group had the highest number of CD68-positive cells at all time points. Figure 3B showed that the control group produced significantly fewer TGF-β1-positive cells than the other two groups on day 0, day 3, and day 5. The pattern is very similar to CD68, in that while the CAPE-T and CAPE-PT groups are not significantly different, CAPE-PT consistently produced more TGF-β1-positive cells (Figure 3B).

**Figure 4:**
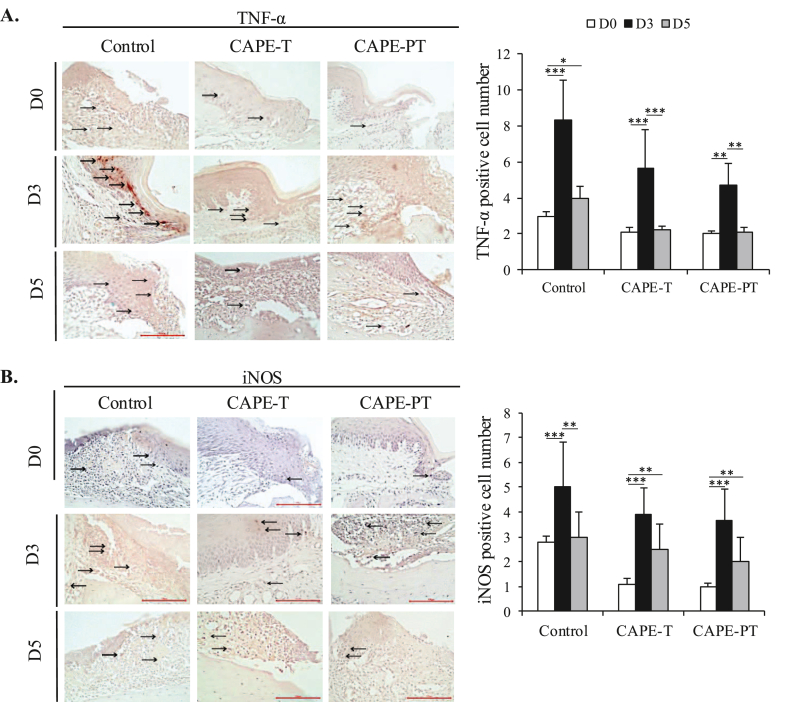
**Expression of IHC Biomarkers TNF-α and iNOS in Palatal Wound Healing.** (A) Representative IHC images and counting of TNF-α-positive cells in the perimeter of the wound site. (B) Representative IHC images and quantification of iNOS-positive cells at the wound site. Scale bar: 100 μm. Data are presented as mean ± SD. ∗∗∗p < 0.001, ∗∗p < 0.01, ∗p < 0.05.

The expression of TNF-α and iNOS, key markers of pro-inflammatory M1 macrophages,^40^ exhibited a dynamic pattern during the wound healing process. In all groups, expression levels peaked on day 3, coinciding with the inflammatory phase of healing, followed by a decline on day 5 as the inflammatory response subsided. Notably, CAPE-treated groups demonstrated a significant reduction in TNF-α and iNOS expression compared to the control group on both day 3 and 5 (Figure 4A, B). This suggests that CAPE modulates the inflammatory response by downregulating pro-inflammatory macrophage activity. Furthermore, inflammatory biomarker analysis revealed that CAPE effectively upregulated CD68 and TGF-β1 expression, indicating an enhanced transition towards the anti-inflammatory M2 macrophage phenotype.^41^ These findings support the hypothesis that CAPE accelerates wound resolution by promoting macrophage polarization and reducing excessive inflammation, ultimately contributing to improved wound healing outcomes.

## Discussion

The present study aimed to investigate the effects of CAPE on palatal wound healing, with a specific focus on macrophage markers expression (CD68, TGF-β1, TNF-α, and iNOS). Given CAPE's established anti-inflammatory, antioxidant, and wound-healing properties, we hypothesised that it could accelerate healing by modulating inflammatory responses. Our findings demonstrate that CAPE enhances palatal wound healing, as evidenced by histological improvements and increased expression of macrophage-associated markers (CD68 and TGF-β1) involved in tissue repair.

CAPE has previously been explored in the context of periodontal disease, dental caries prevention, and inflammation reduction during orthodontic treatment.^42^, ^43^, ^44^, ^45^ Studies also report its efficacy in stimulating epidermal regeneration, decreasing inflammatory cell infiltration, and accelerating wound contraction.^16^^,^^19^, ^20^, ^21^ However, while CAPE has been explored in the context of oral wounds, its specific impact on palatal wound healing and macrophage-related mechanisms remains underexplored. To address this gap, we employed a preclinical murine model where CAPE was administered intraperitoneally at 4 mg/kg body weight, a dosage previously validated in wound healing studies.^29^ This dosage has been shown to exert antioxidant and anti-inflammatory effects, enhancing the elimination of ROS and modulating cytokine production, T-cell proliferation, and lymphokine release.^29^^,^^46^ CAPE inhibits NF-κB activation, contributing to its immunomodulatory properties.^47^ Although we did not directly assess its absorption, pharmacokinetic studies indicate rapid systemic bioavailability following i.p administration.^48^ Future research should further explore CAPE's pharmacokinetics and long-term therapeutic potential.

Interestingly, CAPE administration was associated with significant weight gain in treated mice (Figure 1), consistent with previous studies suggesting its influence on lipid metabolism and fat accumulation.^49^^,^^42^ Histological analysis revealed no significant macrophage infiltration in the liver (Supplementary Figure 1), supporting CAPE's hepatoprotective effects.^45^ However, the underlying mechanisms of these systemic effects warrant further investigation.

In both the CAPE-treated groups, a significant decrease in palatal wound area and EED, along with increase in the percentage of palatal wound closure, was observed compared to controls on days 3 and 5 post-wounding. Notably, the CAPE-PT group exhibited slightly greater wound area reduction than the CAPE-T group, suggesting that pre-treatment may enhance healing outcomes (Figure 2). These findings align with previous studies demonstrating CAPE's role in accelerating re-epithelialization and modulating inflammatory responses during palatal wound healing ^22 30 19^.

CD68 and TGF-β1, key macrophage-associated markers, were significantly elevated in CAPE-treated groups, with the highest expression in the CAPE-PT group (Figure 3). By day 5, when inflammation typically subsides, elevated levels of these markers suggest an active coordination between macrophages and growth factors, promoting tissue remodeling.^21^^,^^50^ Given that CD68-positive macrophages are primary sources of TGF-β1, these findings reinforce CAPE's role in enhancing macrophage activity and growth factor-mediated healing.^8^

CAPE's immunomodulatory effects are further reflected in TNF-α and iNOS expression patterns (Figure 4). These pro-inflammatory markers peaked on day 3 and declined by day 5, consistent with the natural transition from inflammation to tissue regeneration.^51^^,^^52^ Early TNF-α and iNOS upregulation suggests CAPE may initially promote a controlled inflammatory response, facilitating debris clearance and immune activation. The subsequent decline, accompanied by sustained TGF-β1 expression, indicates a shift towards resolution and tissue repair.^27^ This suggests CAPE promotes macrophage polarization toward an M2 phenotype, essential for tissue remodeling.^53^

This study has several limitations. While IHC provides a semi-quantitative assessment of protein expression, future studies should incorporate ELISA or other quantitative methods to measure NO, interleukins, and ROS levels in wound tissue.^54^ Additionally, the absence of an intraclass correlation coefficient (ICC) for wound area measurements is a limitation, despite efforts to minimize errors through repeated evaluations by a calibrated examiner. Incorporating ICC analysis in future research would enhance measurement reliability. Our focus on days 3 and 5 post-wounding primarily captures the inflammatory phase dominated by M1 macrophages. To address this, we included TNF-α and iNOS IHC to characterize M1 macrophage activation. However, this timeframe limits the assessment of the M1-to-M2 transition, which occurs later. Future studies should include later time points and M2 markers (e.g., CD206, Arg-1) to provide a more comprehensive understanding of CAPE's role in macrophage polarization and wound healing.^55^

## Conclusion

Our findings demonstrate that systemic administration of CAPE is associated with a significant increase in body weight, though the underlying metabolic mechanisms remain unclear. Additionally, CAPE positively influences palatal wound healing by accelerating wound closure and modulating CD68, TGF-β1, TNF-α, and iNOS expression. These results suggest that CAPE may have therapeutic potential in enhancing oral wound healing. However, further longitudinal studies are required to fully elucidate CAPE's effects on macrophage polarization and its broader metabolic implications in oral wound management.

## Ethical approval

This study was ethical approved by the Health Research Ethics Commission Faculty of Dentistry, Universitas Islam Sultan Agung, Indonesia (protocol No. 573/B.1-KEPK/SA-FKG/VI/2024).

## Author contributions

IRH contributed to the study's conceptual design, assessed the results, and drafted the article. AOS contributed to analyze the result and drafting of the manuscript. The study was conducted by IRH, AOS, and YB. YSR, IRH, AOS, and YB. The authors made substantial contributions to improved the article's language and style via critical editing, quality assessment, and improvement. IRH and YSR revised the manuscript and approved the final version. Each author has scrutinized and approved the final article and is accountable for its content and similarity index.

## Source of funding

This study was funded by Kementerian Pendidikan, Kebudayaan, Kebudayaan, Riset, dan Teknologi (Kemdikbudristek) through Lembaga Layanan Pendidikan Tinggi (LLDIKTI) (0459/E5/PG.02.00/2024), and distributed by Lembaga Penelitian dan Pengabdian kepada Masyarakat (LPPM) UNISSULA (33/B.1/SA-LPPM/VI/2024).

## Declaration of competing interest

The authors declare that there is no conflict of interest.

## Appendix A Supplementary data

Supplementary 1 Liver size was measured on day 0 and day 5 post-wound creation. (A) Images of the mice livers (scale bar: 10 mm) and HE stained sections examined to assess macrophage appearance in the liver (scale bar: 100 μm). (B) The number of macrophage-positive cells in the liver was quantified on day 0 and day 5 post-wound creation. Scale bar: 100 μm. Data are presented as mean ± SD. ∗∗∗p < 0.001, ∗∗p < 0.01. Supplementary 1
