# Supplementary figures and images for: Caffeic acid phenethyl ester promotes palatal wound healing and enhances wound-associated macrophage CD68 expression
Source: J Taibah Univ Med Sci. 2025 Aug 8;20(4):546–55. doi: 10.1016/j.jtumed.2025.07.010 (PMC12356463; doi:10.1016/j.jtumed.2025.07.010)

Supplementary Figure 1

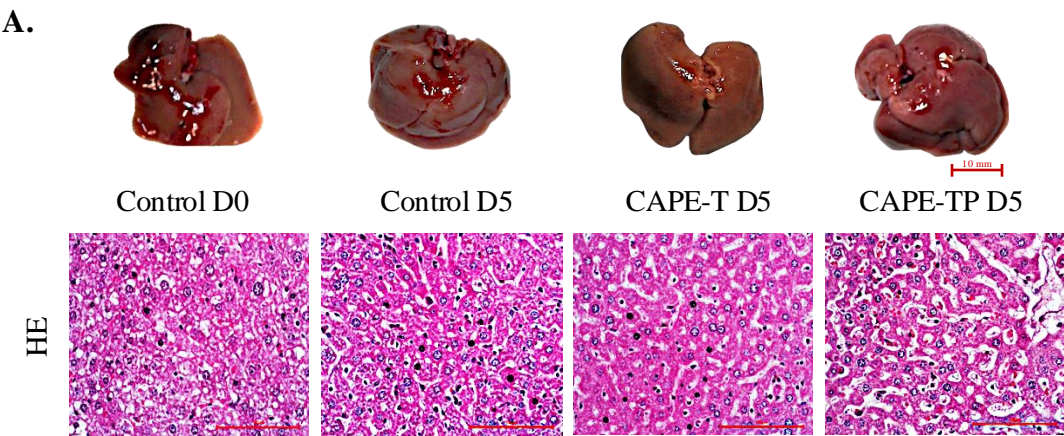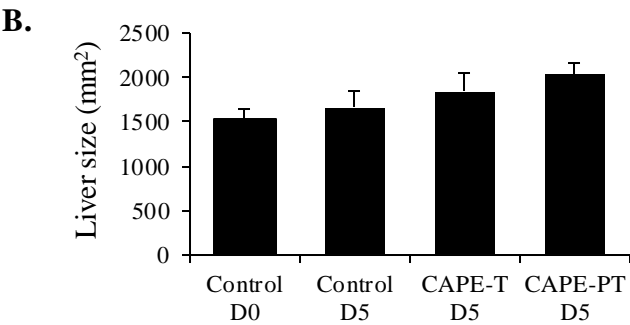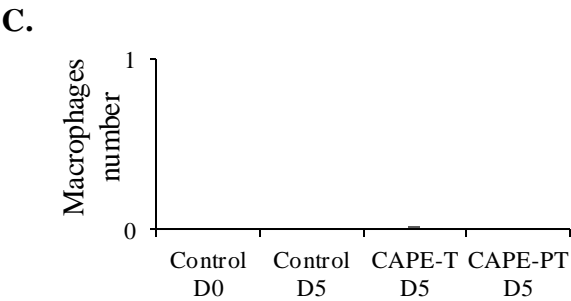

Supplement: Supplementary 1 — Liver size was measured on day 0 and day 5 post-wound creation. (A) Images of the mice livers (scale bar: 10 mm) and HE stained sections examined to assess macrophage appearance in the liver (scale bar: 100 μm). (B) The number of macrophage-positive cells in the liver was quantified on day 0 and day 5 post-wound creation. Scale bar: 100 μm. Data are presented as mean ± SD. ∗∗∗p < 0.001, ∗∗p < 0.01. [file mmc1.pdf]
